# Different Roles for Contracture and Calpain in Calcium Paradox-Induced Heart Injury

**DOI:** 10.1371/journal.pone.0052270

**Published:** 2012-12-20

**Authors:** Jian-Ying Zhang, Wei Tong, Feng Wu, Sheng-Hui Bi, Ming Xu, Zhen-Xiao Jin, Yang Yang, Xiao-Fan Jiang, Jing-Jun Zhou

**Affiliations:** 1 Department of Physiology, Xijing Hospital, The Fourth Military Medical University, Xi’an, China; 2 Department of Out-Patient Clinic, Xijing Hospital, The Fourth Military Medical University, Xi’an, China; 3 Department of Cardiology, Xijing Hospital, The Fourth Military Medical University, Xi’an, China; 4 Department of Cardiovascular Surgery, Xijing Hospital, The Fourth Military Medical University, Xi’an, China; 5 Department of Neurosurgery, Xijing Hospital, The Fourth Military Medical University, Xi’an, China; Albert Einstein College of Medicine, United States of America

## Abstract

The Ca^2+^ paradox represents a good model to study Ca^2+^ overload injury in ischemic heart diseases. We and others have demonstrated that contracture and calpain are involved in the Ca^2+^ paradox-induced injury. This study aimed to elucidate their roles in this model. The Ca^2+^ paradox was elicited by perfusing isolated rat hearts with Ca^2+^-free KH media for 3 min or 5 min followed by 30 min of Ca^2+^ repletion. The LVDP was measured to reflect contractile function, and the LVEDP was measured to indicate contracture. TTC staining and the quantification of LDH release were used to define cell death. Calpain activity and troponin I release were measured after Ca^2+^ repletion. Ca^2+^ repletion of the once 3-min Ca^2+^ depleted hearts resulted in almost no viable tissues and the disappearance of contractile function. Compared to the effects of the calpain inhibitor MDL28170, KB-R7943, an inhibitor of the Na^+^/Ca^2+^ exchanger, reduced the LVEDP level to a greater extent, which was well correlated with improved contractile function recovery and tissue survival. The depletion of Ca^2+^ for 5 min had the same effects on injury as the 3-min Ca^2+^ depletion, except that the LVEDP in the 5-min Ca^2+^ depletion group was lower than the level in the 3-min Ca^2+^ depletion group. KB-R7943 failed to reduce the level of LVEDP, with no improvement in the LVDP recovery in the hearts subjected to the 5-min Ca^2+^ depletion treatment; however, KB-R7943 preserved its protective effects in surviving tissue. Both KB-R7943 and MDL28170 attenuated the Ca^2+^ repletion-induced increase in calpain activity in 3 min or 5 min Ca^2+^ depleted hearts. However, only KB-R7943 reduced the release of troponin I from the Ca^2+^ paradoxic heart. These results provide evidence suggesting that contracture is the main cause for contractile dysfunction, while activation of calpain mediates cell death in the Ca^2+^ paradox.

## Introduction

It is well documented that Ca^2+^ participates in numerous physiological functions in the heart, such as excitation-contraction coupling and excitability [1], [2], whereas abnormalities in Ca^2+^ homeostasis is a common phenomenon that occurs during progressive heart failure [3] and myocardial ischemia/reperfusion injury, i.e., thrombolysis treatment or percutaneous transluminal coronary angioplasty after acute thrombosis formation and restored circulation to the heart following the interruption of flow during open heart surgery [4]. To date, various studies have shown that Ca^2+^ overload leads to mechanical dysfunction, arrhythmias, and cell death [3], [4]. Therefore, it is important to unravel the mechanism in order to learn useful strategies for the prevention of ischemia/reperfusion injury.

The loss of Ca^2+^ homeostasis is easily reproduced by successive perfusion of hearts with Ca^2+^-free and Ca^2+^-containing media in the laboratory, which is termed the Ca^2+^ paradox [5], [6]. One of the most apparent changes after repletion of the once Ca^2+^-depleted hearts is diastolic dysfunction, or the development of contracture, which induces physical stress [7], [8], [9], [10]. This aspect is manifested by the formation of contraction bands, sustained cell shortening, or an elevated left ventricle end-diastolic pressure (LVEDP) in the tracing of left ventricle pressure [7], [8], [9]. The contracture is the accepted primary mediator, and it tears the sarcolemmal membrane apart from adjacent cells, leading to myoglobin, lactate dehydrogenase (LDH) and creatine kinase release, consequently resulting in cell death and heart dysfunction [5], [10]. However, some data cannot be explained by this theory. For example, in cultured cell models, which are free from mechanical interactions with adjacent cells, suppressing the Na^+^/Ca^2+^ exchanger (NCX) with SEA0400 decreased cell death induced by the Ca^2+^ paradox [11]. Therefore, it is possible that other mechanisms are involved in Ca^2+^ paradox-induced heart injury.

**Figure 1:**
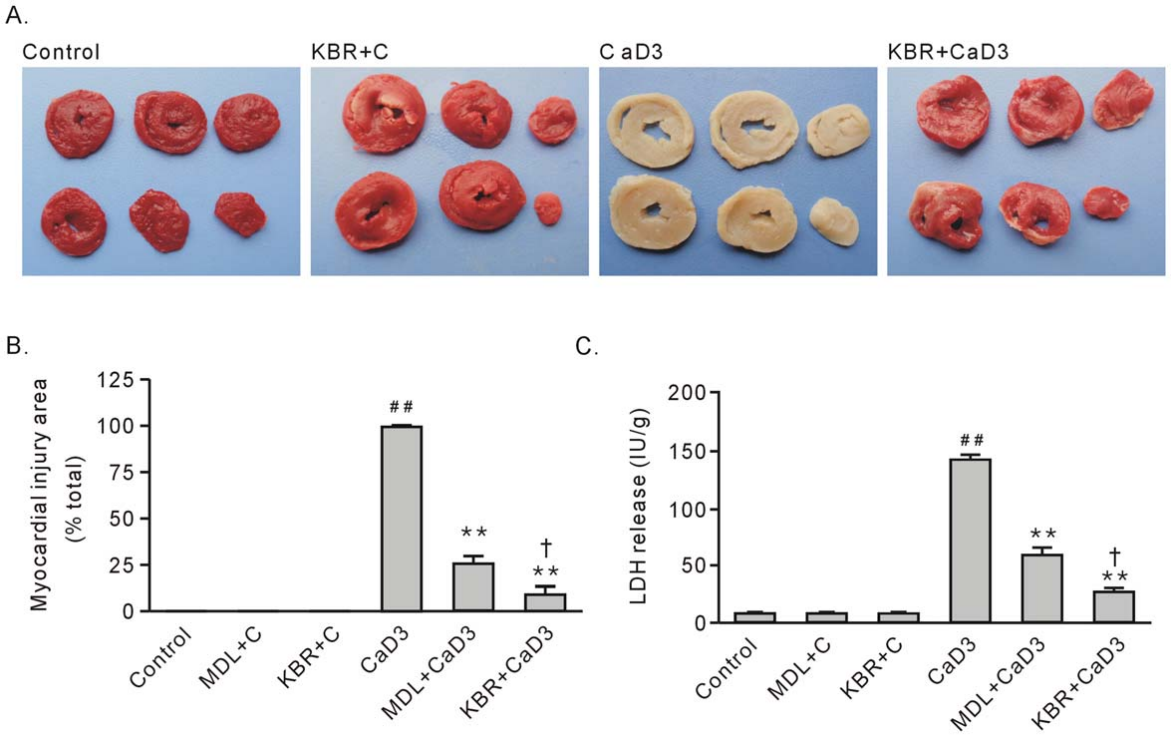
The effects of treatment with 10 µM KB-R7943 or 10 µM MDL28170 on the survival of heart tissue after the 3-min Ca^2+^ depletion treatment. A. Representative TTC staining for hearts subjected to the 3-min Ca^2+^ depletion treatment in the absence or presence of KB-R7943. TTC-positive staining represents viable tissue. B. Group results on the myocardial injury area, which is expressed as a percentage of the total area. C. Group results on LDH release. Each bar represents the mean±SEM, n = 6. ^##^ *P*<0.01 vs. the control, **P<0.01 vs. CaD3, ^†^ *P*<0.01 vs. MDL+CaD3. Abbreviations: MDL+C, MDL28170 control; KBR+C, KB-R7943 control; CaD3, 3-min Ca^2+^ depletion group; MDL, MDL28170; and KBR, KB-R7943.

Calpains are intracellular cysteine proteases involved in numerous physiological and pathological phenomena, such as cell migration during wound closure or tumor invasion [12], [13]. Among the 15 members of the calpain family, the two best characterized calpains, known as μ-calpain and m-calpain, are expressed in the myocardium [13], [14], [15]. Although the amount of Ca^2+^ required for the in vitro activation of μ-calpain and m-calpain was different and m-calpain was regulated by binding to phosphatidylinositol 4,5-bisphosphate [12], [13], ample studies have shown that calpains are activated during ischemia/reperfusion, resulting in the cleavage of its substrates, such as Na^+^/K^+^-ATPase, α-fodrin, a prominent component of the membrane skeleton, and consequently heart injury [16], [17], [18], [19]. We and others have previously found that after Ca^2+^ repletion, calpains are activated and both μ- and m-calpain are anchored to the sarcolemmal membrane [20], [21]; moreover, the addition of MDL28170, an inhibitor of calpain, reduced the cleavage of α-fodrin and rescued myocardial dysfunction and cell death [20]. However, MDL28170 did not substantially reduce the level of LVEDP and the degradation of troponin I [20], a regulatory protein involved in maintaining the diastolic state. Based on these results, we hypothesized that calpain activation and contracture development were two independently important events in the cascade of the Ca^2+^ paradox, which resulted in multiple cell abnormalities that led to the heart injury.

In this study, KB-R7943, a selective inhibitor of NCX [22], [23], and MDL28170, an inhibitor of calpain, were used. We firstly compared the effects of KB-R7943 and MDL28170 on the recovery of LVEDP, an indicator of the contracture, in hearts subjected to the 3-min Ca^2+^ depletion treatment; we secondly analyzed the relationship between the contracture and contractile function recovery and between the contracture and tissue survival in the Ca^2+^ paradox. Thirdly, we prolonged the duration of Ca^2+^ depletion to 5 min to reduce the diminishing effects of KB-R7943 on the LVEDP and then evaluated its effects on the contractile function recovery and survival of tissue after Ca^2+^ repletion. Finally, we determined the effects of KB-R7943 and MDL28170 on calpain activity and troponin I release. These results suggest that contracture is the main cause for contractile dysfunction, while activation of calpain mediates cell death in the Ca^2+^ paradox.

**Figure 2:**
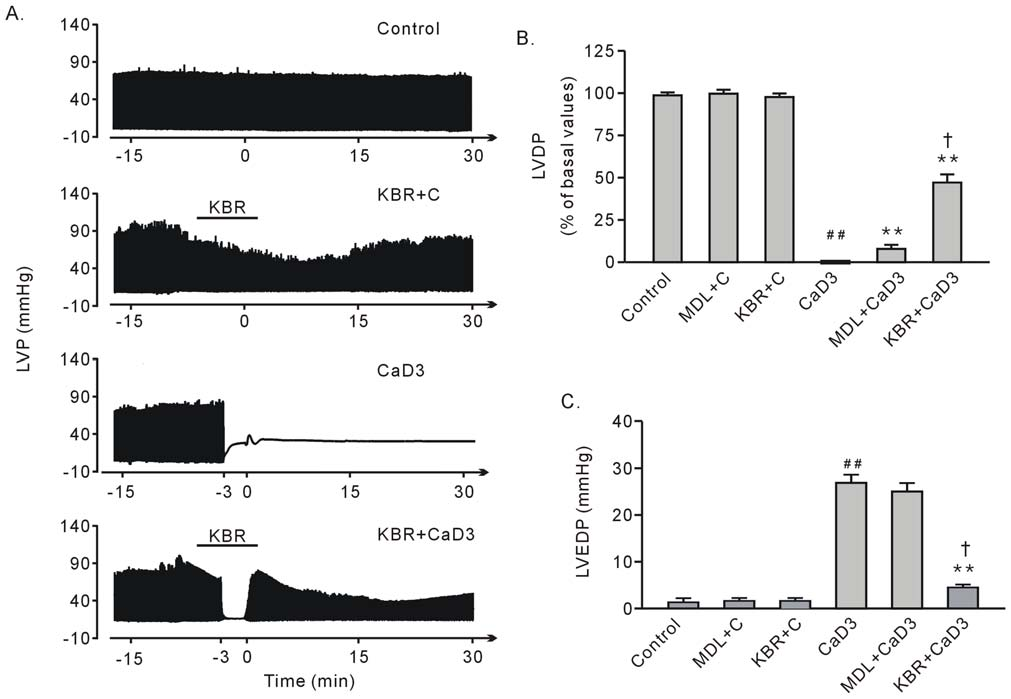
The effects of treatment with 10 µM KB-R7943 or 10 µM MDL28170 on the recovery of heart function after the 3-min Ca^2+^ depletion treatment. A. Representative left ventricular pressure tracing in the hearts subjected to the 3-min Ca^2+^ depletion treatment in the presence or absence of KB-R7943. B. Group results on the recovery of LVDP at the end of perfusion. C. Group results on LVEDP at the end of perfusion. Each bar represents the mean±SEM, n = 6. ^##^ *P*<0.01 vs. the control, **P<0.01 vs. with CaD3, ^†^ *P*<0.01 vs. MDL+CaD3. Please refer to Figure 1 for the abbreviations.

## Materials and Methods

### Drugs and Chemicals

KB-R7943 and MDL28170 were purchased from Tocris Bioscience (Ellisville, MO, USA). The LDH enzyme-linked immunoassay (ELISA) kit was purchased from R&D Systems (Minneapolis, MN, USA). Rabbit anti-μ-calpain and -m-calpain antibodies were obtained from Cell Signaling Technology (Beverly, MA, USA). Mouse anti-α-fodrin antibody was purchased from Enzo Life Sciences (Plymouth Meeting, PA, USA). A tetramethylrhodamine goat anti-rabbit IgG was purchased from Molecular Probes (Eugene, OR, USA). Triphenyltetrazolium chloride (TTC), 4′,6-Diamidino-2-phenylindole dihydrochloride (DAPI), Ponceau Red, and other chemicals were from Sigma (Shanghai, China).

### Animals

Sprague-Dawley male rats of 250∼300 g body weight were obtained from the Laboratory Animal Centers at The Fourth Military Medical University. All experimental procedures described here were in accordance with the National Institutes of Health’s Guide for the Care and Use of Laboratory Animals and were approved by the Institutional Animal Care and Use Committee at The Fourth Military Medical University.

**Figure 3:**
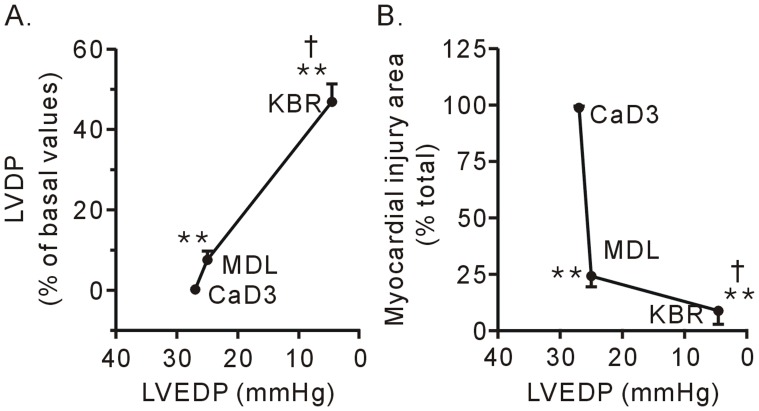
The contractile function recovery (A) and improvement in cell survival (B) after treatment with 10 µM KB-R7943 (KBR) was correlated to the recovery of LVEDP in the hearts subjected to the 3-min Ca^2+^ depletion treatment. Each bar represents the mean±SEM, n = 6. **P<0.01 vs. the 3-min Ca^2+^ depletion group (CaD3), ^†^ *P*<0.01 vs. MDL28170 (MDL).

### Langendorff-perfused Rat Heart

As described previously [20], the hearts were quickly removed and retrogradely perfused through the aorta in a non-circulating Langendorff apparatus (Radnoti Glass Technology Inc., Monrovia, CA, USA) at a constant pressure of 80 mm Hg. Krebs-Henseleit (KH) was used as a perfusion solution and contained 118 mM NaCl, 4.7 mM KCl, 1.2 mM MgSO_4_, 1.2 mM KH_2_PO_4_, 1.25 mM CaCl_2_, 25 mM NaHCO_3_, and 11 mM glucose (pH 7.4, 37°C). The solution was equilibrated with 95% O_2_/5% CO_2_. A water-filled latex balloon-tipped catheter was carefully placed into the left ventricle through the left atrium and adjusted to a left ventricular end-diastolic pressure (LVEDP) of approximately 5 mm Hg by inflating the balloon. The distal portion of the catheter was connected to a computer via a transducer (Model 100 BP-Biopac System Inc., Goleta, CA, USA). Data processing was performed using AcqKnowledge software (version 3.8.1). The index of myocardial contractile function was determined with the left ventricular developed pressure (LVDP), which was calculated from the difference between the left ventricular peak systolic pressure and end-diastolic pressure. The LVEDP was used to reflect the diastolic function, or the degree of contracture occurring at the end of the experiment. A 10-min equilibration period was allowed before the experiments started.

**Figure 4:**
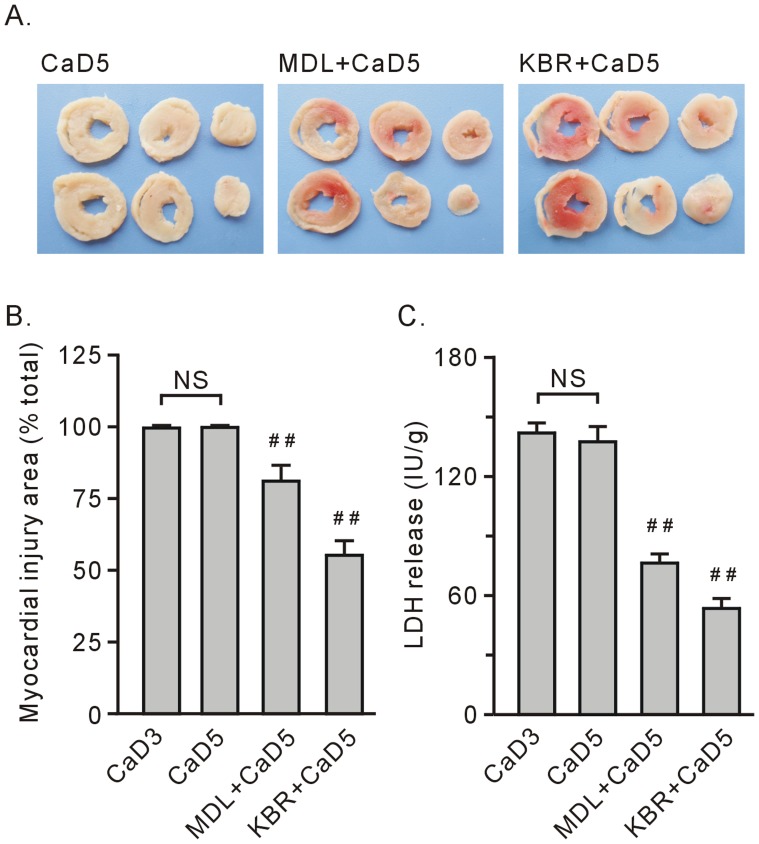
Treatment with 10 µM KB-R7943 (KBR) or 10 µM MDL28170 (MDL) preserved heart tissue survival after the 5-min Ca^2+^ depletion treatment. A. Representative heart tissue slice stained with TTC. B. Group results on the myocardial injury area, which was expressed as a percentage of the total area. C. Group results on LDH release. Each bar represents the mean±SEM, n = 6. ^##^ *P*<0.01 vs. the 5-min Ca^2+^ depletion group (CaD5). NS stands for no significant difference between the 3-min Ca^2+^ depletion group (CaD3) and CaD5.

### Study Groups and Experimental Protocol

The experiments were randomly divided into 9 groups. Every group had 6 hearts. For the normal control (Group 1), the hearts were perfused for 45 min with normal KH solution. For the drug control (Group 2 and 3), the hearts were treated with 10 µM KB-R7943 or MDL-28170 for 8 min followed by perfusion with normal KH solution. In the 3-min Ca^2+^ depletion protocol, the heart was successively perfused with Ca^2+^-free KH solution for 3 min and KH solution containing 1.25 mM Ca^2+^ for 30 min without (Group 4) or with KB-R7943 (Group 5) and MDL28170 (Group 6). In the 5-min Ca^2+^ depletion protocol, the heart was depleted of Ca^2+^ for 5 min without (Group 7) or with KB-R7943 (Group 8) and MDL28170 (Group 9).

**Figure 5:**
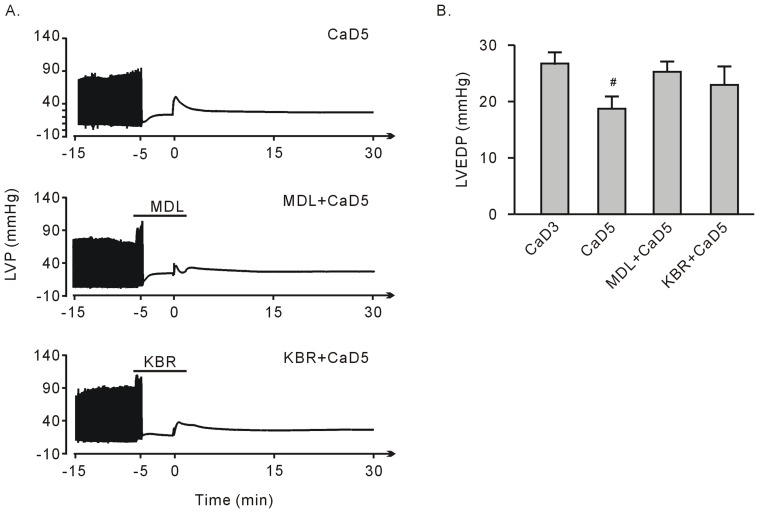
Treatment with 10 µM KB-R7943 (KBR) or 10 µM MDL28170 (MDL) failed to rescue heart function recovery after the 5-min Ca^2+^ depletion treatment. A. Representative left ventricular pressure tracing. B. Group results on the LVEDP at the end of perfusion. Each bar represents the mean±SEM, n = 6. Compared to the 3-min Ca^2+^ depletion group (CaD3), ^#^ *P*<0.05.

For the Ca^2+^-free KH solution, Ca^2+^ was omitted, and 0.1 mM EDTA was added to ensure the removal of any contaminating Ca^2+^. A 10 µM concentration of KB-R7943 or MDL28170 was added during the 1-min perfusion prior to Ca^2+^ depletion, during the entire period of Ca^2+^ depletion, and for the first 2 min of Ca^2+^ repletion. The concentrations of KB-R7943 and MDL28170 used in this study were based on previous studies [23], [24], [25], [26].

### Myocardial Infarct Size Determination

At the end of experiments, the heart was frozen, cut into slices perpendicular to the apex-base axis, incubated in 1.0% TTC for 20 min at 37°C, and then immersed in 10% formalin overnight. Viable myocardium was stained brick red and the absence of TTC staining demarcated the areas of irreversibly injured myocardium. The area of myocardial injury was determined by a computerized planimetry technique (OPTIMAS v. 5.2, BioScan Inc., Edmonds, WA, USA) and expressed as a percentage of the total area [20].

**Figure 6:**
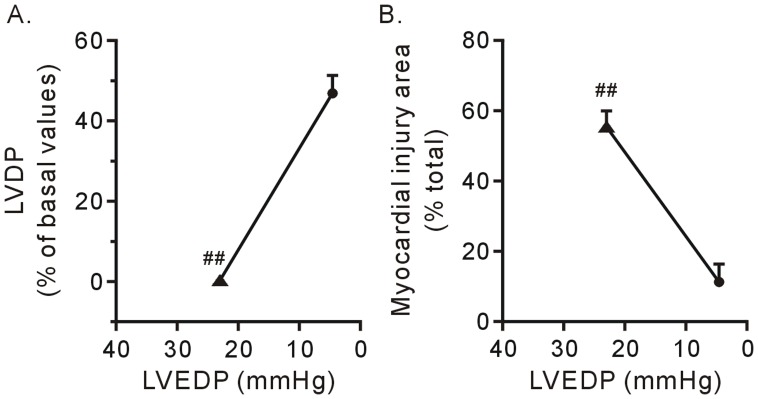
Impairment of contractile function recovery (A) and tissue survival (B) by KB-R7943 was correlated to its blunted effects on LVEDP in the hearts subjected to the 5-min Ca^2+^ depletion. Each bar represents the mean±SE, n = 6. The circle refers to the 3-min Ca^2+^ depletion group, and the triangle refers to the 5-min Ca^2+^ depletion group; ^##^ *P*<0.01 vs. the 3-min Ca^2+^ depletion group.

### LDH and Troponin I Release Assay

The coronary effluent was collected during the 30 min of Ca^2+^ repletion, and LDH, an indicator of cell death [27], [28], was measured by an ELISA kit (R&D Systems, Minneapolis, MN, USA). The amount of LDH released during the 30 min of Ca^2+^ repletion was normalized against the wet weight of the heart and expressed as IU/g.

Cardiac troponin I in the coronary effluent was measured with the current version of the AccuTnI assay (Beckman Coulter Inc, Fullerton, CA, USA). The Access Immunoassay System is a random access, bench-top analyzer that has been described in detail in previous studies [29], [30].

### In vitro Calpain Activity Assay

The frozen left ventricle tissue was homogenized in an ice-cold Tris-buffered saline solution containing 20 mM Tris-HCl (pH 7.3), 1 mM EGTA, 150 mM NaCl, and 1% Triton X-100 and centrifuged for 15 min at 15,000×g. The supernatant was collected and stored at −70°C until use. Calpain activity was measured by fluorometry (BioTek Synergy HT Microplate Reader) using Suc-LLVY-AMC (Calbiochem) as a substrate, as previously described [16], [17]. The release of the fluorescent product AMC was assessed using excitation and emission wavelengths of 380 nm and 460 nm, respectively. The calpain inhibitor MDL28170 at a concentration of 10 µM was used to determine the specificity of the assay.

**Figure 7:**
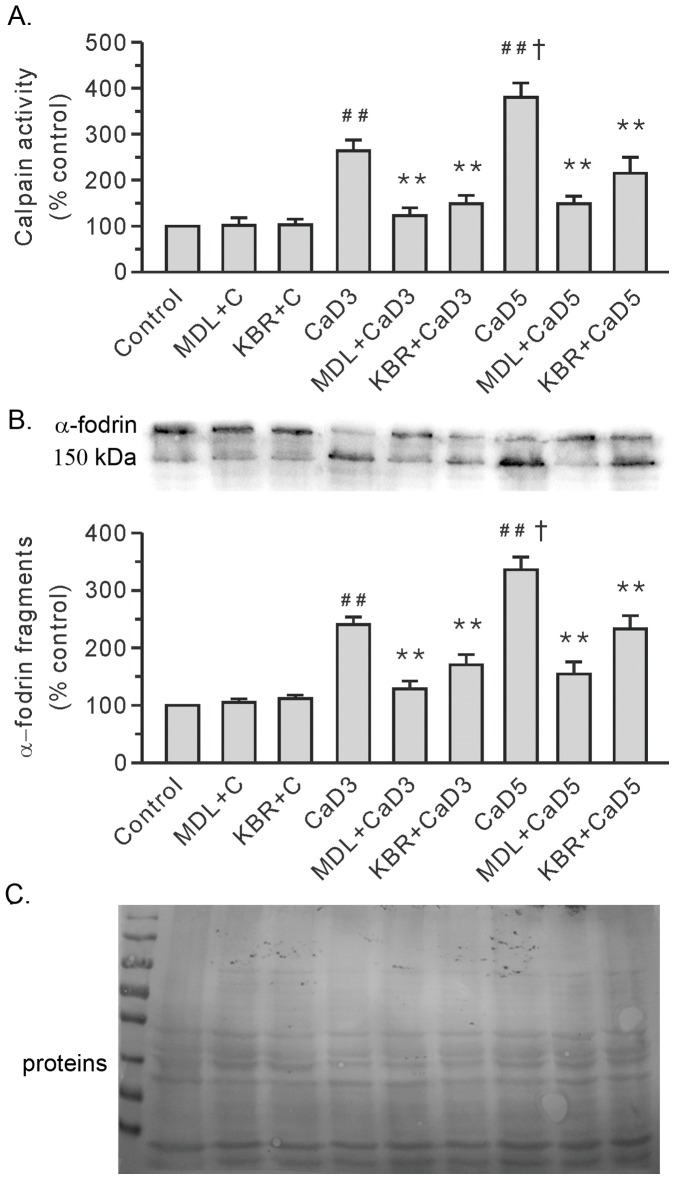
Treatment with 10 µM KB-R7943 (KBR) or 10 µM MDL28170 (MDL) reduced the activity of calpain in the Ca^2+^ paradoxic hearts. A. Group results on calpain activity by using Suc-LLVY-AMC. B. Representative blots for α-fodrin are shown at the top, along with the densitometric analysis result (bottom). C. Ponceau Red staining of the membrane. The values were expressed as arbitrary units relative to the control, which was given a value of 1. Each bar represents the mean±SEM, n = 6. ^##^ *P*<0.01 vs. the control, ^†^ *P*<0.01 vs. the 3-min Ca^2+^ depletion group (CaD3), **P<0.01 vs. the corresponding group without the drug. MDL+C, MDL28170 control; KBR+C, KB-R7943 control; and CaD5, 5-min Ca^2+^ depletion group.

### Western Blot Analysis

Two minutes after Ca^2+^ repletion, the heart was removed, and the left ventricular myocardium was frozen in liquid nitrogen and stored at −70°C until use. Approximately 100 mg of frozen tissue was homogenized, using a Bio-Gen PRO200 homogenizer, for 30 s at 4°C in 0.5 ml of lysis buffer containing 50 mM Tris-HCl (pH 7.3), 150 mM NaCl, 5 mM EDTA, 1 mM dithiothreitol, with 1% Triton X-100 and 1% protease inhibitor cocktail. The lysates were centrifuged for 15 min at 12,000×g, and the resulting supernatant was aliquoted and stored at −70°C. Total protein concentrations were determined using the Bradford protein assay kit, and 40 µg of the protein was used to evaluate the calpain activity by Western blot using a monoclonal antibody against α-fodrin, which recognizes the calpain-cleaved fragment (approximately 150 kDa), as described previously [18], [31]. The samples were resolved using an 8% SDS-polyacrylamide gel and transferred onto nitrocellulose membranes. The membranes were incubated with a primary antibody against α-fodrin. An anti-mouse IgG peroxidase conjugate was used as a secondary antibody. The protein bands were detected by chemiluminescence and quantified using the Image Lab software package (Bio-Rad Laboratories, Herts, UK). The membranes were stained with Ponceau Red to confirm that equal amounts of protein were loaded.

**Figure 8:**
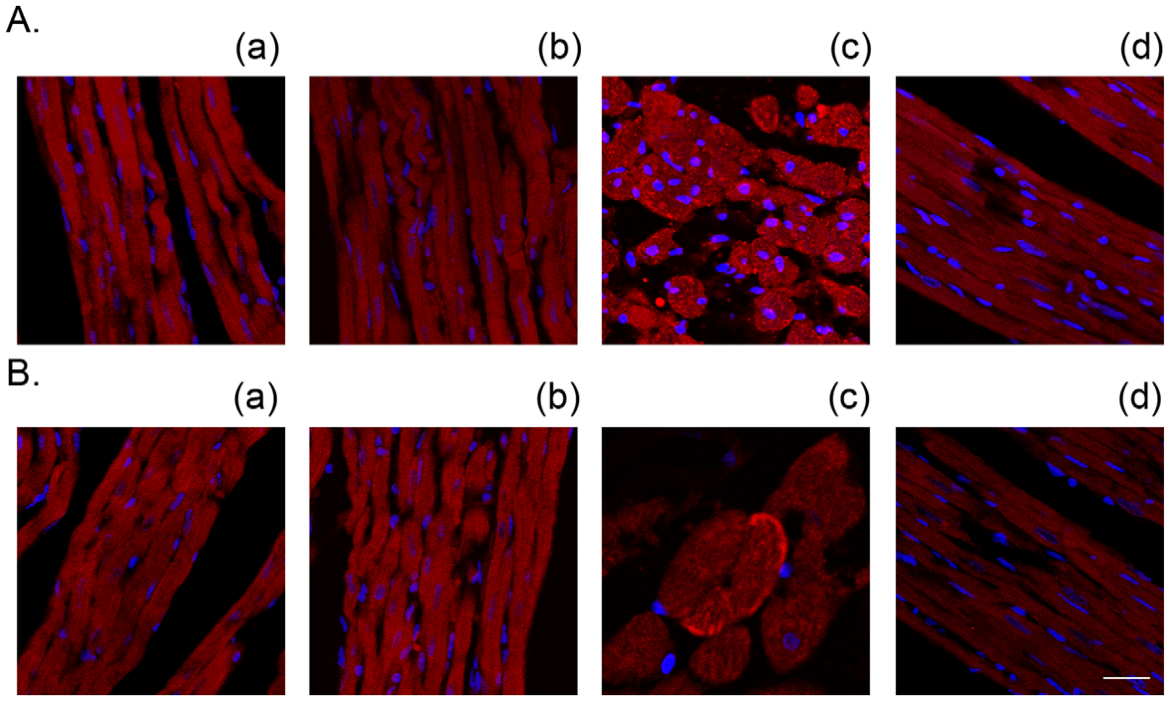
Treatment with 10 µM KB-R7943 attenuated the Ca^2+^ repletion-induced membrane anchoring of both μ- (A) and m-calpain (B) in the 3-min Ca^2+^ depletion group. The nuclei were counterstained with DAPI (blue). (a) control, (b) KB-R7943 control, (c) Ca^2+^ paradox, (d) Ca^2+^ paradox with KB-R7943. Scale bar, 5 µm.

### Immunofluorescence Histochemistry and Confocal Analysis

Two minutes after Ca^2+^ repletion, the left ventricle tissue was excised, fixed in 4% paraformaldehyde at 4°C overnight, embedded in paraffin, and cut into 3-µm-thick sections. After deparaffinization and rehydration, the sections were stained with anti-μ-calpain (1∶100) and anti-m-calpain antibody (1∶25) at 4°C overnight, followed by a 60-min incubation at room temperature with a TRITC-labeled goat anti-rabbit IgG second antibody. DAPI was used to label the nuclei. The sections were examined using a laser-scanning confocal microscope equipped with the FV10-ASW system (Olympus FV1000). The images were analyzed using Image-Pro Plus 5.0 software (Media Cybernetics Inc, Rockville, MD 20850, USA) to quantify the percentage of membrane-anchored calpain, as described previously [32]. Briefly, the sarcolemmal membrane sheets were individually outlined, and the average pixel intensity within each sheet was determined. The background intensity was determined using cytosolic areas. The ratios between the membranes and the background above 120% were considered positive results. At least 300 sarcolemmal membrane sheets were examined for each experimental condition by individuals unaware of the group designations.

**Table 1:** The effects of treatment with 10 µM KB-R7943 or 10 µM MDL28170 on the percentage of calpain membrane-positive cells in the hearts subjected to the 3-min Ca^2+^ depletion and 2-min Ca^2+^ repletion.

	control	MDL+C	KBR+C	CaD3	MDL+CaD3	KBR+CaD3
% μ-calpain positive cells	0	0	0	23.2±3.1##	7.6±1.1**	3.5±0.4**
% m-calpain positive cells	0	0	0	3.1±0.4##	2.2±0.5	0.8±0.1**

The data are documented as the mean±SEM, n = 4.

## *P*<0.01 vs. the control,

** *P*<0.01 vs. the corresponding group without the drug. KBR+C, KB-R7943 control; MDL+C, MDL28170 control; CaD3, 3-min Ca^2+^ depletion group; MDL, MDL28170; and KBR, KB-R7943.

### Statistical Analysis

All data are presented as the mean 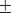 SEM. Group comparisons were performed using ANOVA followed by the Tukey’s test. Statistical analyses were performed using GraphPad Prism v. 4 (GraphPad software, Inc., La Jolla, CA, USA). Two-tailed *P*<0.05 was considered statistically significant.

**Figure 9:**
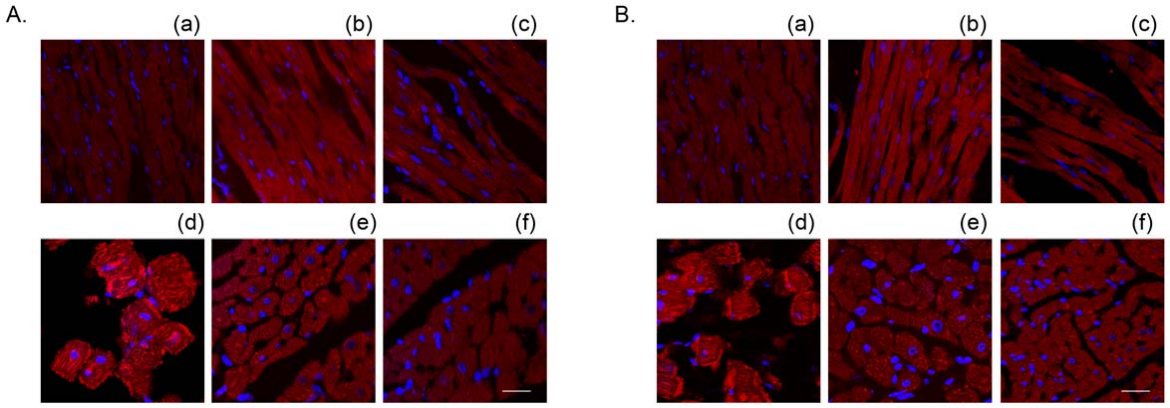
Treatment with 10 µM KB-R7943 or 10 µM MDL28170 attenuated the Ca^2+^ repletion-induced membrane anchoring of both μ- (A) and m-calpain (B) in the 5-min Ca^2+^ depletion group. (a)–(c) represent the control, MDL28170 and KB-R7943 control hearts, respectively, (d)-(f) represent the 5-min Ca^2+^ depletion, with MDL28170 and with KB-R7943, respectively. Scale bar, 5 µm.

## Results

### KB-R7943 Rescued Heart Contractile Function and Tissue Survival after a 3-min Ca^2+^ Depletion More Effectively than MDL28170, with a Marked Drop in LVEDP

As we had stated previously [20], the 3-min Ca^2+^ depletion resulted in no viable tissue, as shown by TTC staining, and an increase in LDH release compared with the control group (Figure 1, A–C). Treatment with 10 µM KB-R7943, administered 1 min before the Ca^2+^ depletion, during the Ca^2+^ depletion, and the first 2 min after the Ca^2+^ repletion almost completely abolished the tissue injury by the 3-min Ca^2+^ depletion treatment, with a small myocardial injury area marked by TTC staining and significant decreases in the release of LDH (Figure 1, A–C).

After Ca^2+^ repletion, the heart exhibited no contractile ability, characterized by diminishing LVDP. The LVEDP significantly increased at the end of the perfusion, suggesting contracture had occurred. Treatment with 10 µM KB-R7943 significantly rescued heart contractile function with LVEDP dropping in level (Figure 2, A–C). Finally, LVDP decreased with KB-R7943 treatment; however, it returned to normal levels after the drug was washed out (Figure 2, A and B).

**Table 2:** The effects of treatment with 10 µM KB-R7943 or 10 µM MDL28170 on the percentage of calpain membrane-positive cells in the hearts subjected to the 5-min Ca^2+^ depletion and 2-min Ca^2+^ repletion.

	control	MDL+C	KBR+C	CaD3	CaD5	MDL+CaD5	KBR+CaD5
% μ-calpain positive cells	0	0	0	23.2±3.1##	34.1±4.5##	11.6±1.5**	5.5±0.8**
% m-calpain positive cells	0	0	0	3.1±0.4##	40.1±5.5##, †	12.3±1.4**	8.2±0.7**

The data are documented as the mean±SEM, n = 4.

## *P*<0.01 vs. the control,

† *P*<0.01 vs. CaD3,

** *P*<0.01 vs. the corresponding group without the drug. KBR+C, KB-R7943 control; MDL+C, MDL28170 control; CaD3, 3-min Ca^2+^ depletion group; CaD5, 5-min Ca^2+^ depletion group; MDL, MDL28170; and KBR, KB-R7943.

Compared to the calpain inhibitor MDL28170, KB-R7943 strongly decreased the level of LVEDP, which was well correlated with improved contractile function recovery (Figure 2 and 3A) and tissue survival (Figure 1 and 3B). These results confirmed that contracture is the main cause for heart injury in the Ca^2+^ paradox. Moreover, the results suggest that, in contrast to calpain, contracture is responsible for contractile dysfunction in the Ca^2+^ paradox.

### KB-R7943 Failed to Rescue Contractile Function after the 5-min Ca^2+^ Depletion but Still Preserved Surviving Tissue, Although Failing to Reduce the LVEDP

Compared to the 3-min Ca^2+^ depletion treatment, repletion of the once 5-min Ca^2+^ depleted hearts did not impose any obvious alterations in TTC staining and LDH release at the end of experiment (Figure 1 and 4). Treatment with KB-R7943 or 10 µM MDL28170 still significantly preserved surviving tissue, although these effects became weakened (Figure 4, A–C).

After repletion of the once 5-min Ca^2+^ depleted hearts, there was no contractile performance as indicated by the vanishing LVDP, a finding similar to that of the 3-min Ca^2+^ depletion treatment (Figure 2 and 5A). The LVEDP significantly increased at the end of perfusion, although it was below the level after the 3-min Ca^2+^ depletion (Figure 5B). Treatments with either 10 µM KB-R7943 or 10 µM MDL28170 failed to rescue the LVDP, with no alterations in the LVEDP (Figure 5, A and B).

Figure 6 shows the relationships between the contracture and contractile function recovery and between the contracture and surviving tissue in the hearts subjected to different durations of Ca^2+^ depletion. In the hearts subjected to the 3-min Ca^2+^ depletion treatment, treatment with 10 µM KBR7943 reduced the LVEDP back to normal levels during Ca^2+^ repletion and significantly improved the contractile function recovery of the heart. In contrast, treatment with 10 µM KBR7943 failed to reduce the LVEDP, and the effects on contractile function recovery and tissue survival were diminished in the hearts subjected to the 5-min Ca^2+^ depletion treatment (Figure 6, A and B).

**Figure 10:**
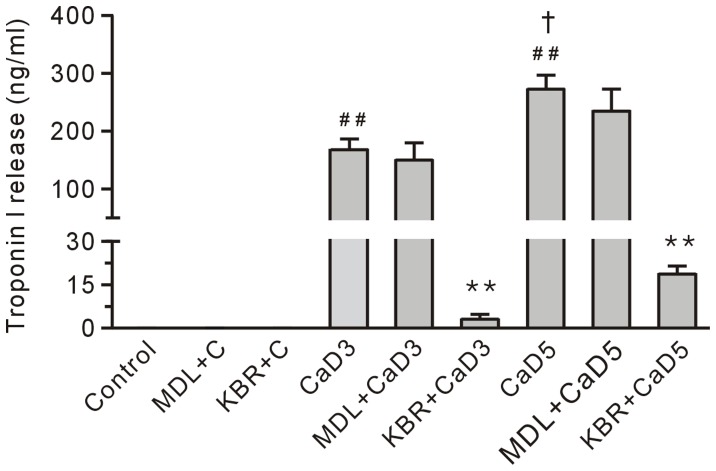
Treatment with 10 µM KB-R7943 (KBR), but not 10 µM MDL28170 (MDL) reduced the level of troponin I in the coronary effluent from the Ca^2+^ paradoxic hearts. Each bar represents the mean±SEM, n = 6. ^##^ *P*<0.01 vs. the control, ^†^ *P*<0.01 vs. the 3-min Ca^2+^ depletion group (CaD3), **P<0.01 vs. the corresponding group without the drug. MDL+C, MDL28170 control; KBR+C, KB-R7943 control; and CaD5, 5-min Ca^2+^ depletion group.

### Both MDL28170 and KB-R7943 Attenuated Ca^2+^ Repletion-induced Increases in Calpain Activity in the 3-min and 5-min Ca^2+^ Depleted Hearts

We measured the calpain activity to delineate the role of calpain in the heart injury induced by the Ca^2+^ paradox. Compared to the control group, repletion of the once 3-min Ca^2+^ depleted hearts induced an increase in calpain activity (Figure 7A). Moreover, the hearts in the 5-min Ca^2+^ depletion group exhibited higher levels of calpain activity than the levels in the 3-min Ca^2+^ depleted group (Figure 7A). The documented cleavage of α-fodrin, a well-known calpain substrate, further substantiated the activation of calpain (Figure 7B). More importantly, KB-R7943, like MDL28170, significantly attenuated Ca^2+^ repletion-induced increases in calpain activity in the 3-min and 5-min Ca^2+^ depleted hearts (Figure 7). Both KB-R7943 and MDL28170 had no effect on the control hearts.

We previously illustrated that repletion of the once 3-min Ca^2+^ depleted hearts induced an increase in membrane-anchored calpain [20]. Therefore, we evaluated the effects of KB-R7943 by confocal imaging. Similar to the calpain inhibitor MDL28170, treatment with 10 µM KB-R7943 blocked the alterations of both μ- and m-calpain, confirming that activation of calpain is an important factor contributing to heart injury by the Ca^2+^ paradox (Figure 8 and Table 1). In contrast to the hearts in the 3-min Ca^2+^ depletion group where μ-calpain was predominantly activated (Figure 8), both μ- and m-calpain were anchored to the sarcolemmal membrane in the hearts in the 5-min Ca^2+^ depletion group (Figure 9 and Table 2), reflecting increased activity of calpain in the 5-min Ca^2+^ depletion group. Nevertheless, these effects were attenuated by both KB-R7943 and MDL28170 (Figure 8 and 9, Table 1 and 2). Neither KB-R7943, nor MDL28170 had an effect in the control hearts (Figure 8 and 9, Table 1 and 2).

### KB-R7943, but not MDL28170, Reduced Ca^2+^ Repletion-induced Troponin I Release in the 3-min and the 5-min Ca^2+^ Depleted Hearts

Compared to the control group, the level of troponin I in the coronary effluent in the 3-min Ca^2+^ depletion group was significantly increased (Figure 10), suggesting that the loss of troponin I may result in the development of a contracture. Furthermore, the amount of troponin I in the coronary effluent in the 5-min Ca^2+^ depletion group was higher than the level in the 3-min Ca^2+^ depletion group (Figure 10), which was consistent with the data showing that the hearts in the 5-min Ca^2+^ depletion group had more severe diastolic dysfunction and contracture (Figure 5). Treatment with 10 µM KB-R7943 significantly reduced the loss of troponin I in the Ca^2+^ paradoxic heart; however, treatment with 10 µM MDL28170 had no effect (Figure 10), indicating calpain was not involved in the development of the contracture in the Ca^2+^ paradoxic heart.

## Discussion

It is generally accepted that the development of the contracture is the primary event in the Ca^2+^ paradox that leads to cell death and heart dysfunction [5], [10]. The most interesting findings in the present study were 1) compared with the calpain inhibitor MDL28170, KB-R7943 was more effective at rescuing heart contractile dysfunction and tissue death after the 3-min Ca^2+^ depletion, and this was accompanied by reduced LVEDP levels to a greater extent; 2) After prolonging the duration of Ca^2+^ depletion to 5 min, KB-R7943 failed to decrease the level of LVEDP and rescue contractile dysfunction, but the treatment still preserved surviving tissue against Ca^2+^ repletion and inhibited calpain activity. These results provide, for the first time, evidence suggesting that contracture is the main cause for contractile dysfunction, and activation of calpain mediates cell death in the Ca^2+^ paradox.

In the current study, we found that both KB-R7943 and MDL28170 improved the survival of heart tissue from the 3-min and 5-min Ca^2+^ depletion treatment, which was accompanied with a reduction in the spike in calpain activity and the degradation of α-fordin. These results support the notion that the activation of calpain is a critical event leading to tissue death in the Ca^2+^ paradox. Furthermore, our data, consistent with previous studies, identified membrane-anchored μ- and m-calpain in the Ca^2+^ paradoxical hearts, suggesting that this step triggers a strong activation of this protease [12], [18]. Thus, it would be interesting to identify the function of calpain and the underlying activation mechanism. In addition, previous studies showed that mitochondria become swollen in shape and functionally defective in the Ca^2+^ paradox [8], [33]. Further studies are needed to clarify the relationship between calpain and proteins related to mitochondria in the Ca^2+^ paradox because mitochondria controls the production of high energy phosphate and cell death, which are regulated by Ca^2+^ [34].

Contracture developing in the Ca^2+^ paradox include the following major characteristics: 1) abnormalities in the sarcomere structure, i.e., some degree of A-band compression and apparent thickening of the Z-bands; 2) contracture force development, which is characterized by an increase in the LVEDP performance and consequently disruption of the sarcolemmal membrane [5], [7], [8], [9], [10]. In the present study, it was found that the intensity of the contracture in the 5-min Ca^2+^ depletion group is weaker than the contracture intensity in the 3-min Ca^2+^ depletion group. This lesser intensity may be due to the amount of Ca^2+^ in the sarcoplasmic reticulum is decreased by the prolonged Ca^2+^-free superfusion time [35]. More importantly, our data revealed that there was a positive relationship between contractile function recovery and the level of LVEDP (Figure 3 and 6), suggesting that contracture results in contractile dysfunction in the Ca^2+^ paradox. Compared to MDL 28710, an inhibitor of calpain, KB-R7943 had greatly decreased the level of LVEDP and improved cell survival. We interpret from our results that force stress from the contracture and the activation of calpain are two key nodes in the Ca^2+^ paradox cascade, which results in hearts injury.

An interesting observation in this study is that KB-R7943 rescued contractile dysfunction after the 3-min Ca^2+^ depletion, which was accompanied with a decreased release of troponin I, a regulatory protein involved in maintaining the diastolic state [36], [37]. This is in contrast to previous studies showing that nifedipine or verapamil had no effects on the recovery of mechanical activity [38]and the data showing that the canonical transient receptor potential channels mediate Ca^2+^ influx [35]. Our data support the view that NCX mediates Ca^2+^ overload in the Ca^2+^ paradox [10], [39]. More importantly, our result suggests that loss of troponin I is an important step for inducing the development of the contracture in the Ca^2+^ paradoxic hearts. KB-R7943 failed to rescue heart contractile dysfunction after the 5-min Ca^2+^ depletion treatment, compared to the corresponding data in the 3-min Ca^2+^ depletion group. The data measuring the level of troponin I in the coronary effluent showed that, although KB-R7943 reduced the release of troponin I in the Ca^2+^ paradoxic hearts, the amount of troponin I release in the 5-min Ca^2+^ depletion with KB-R7943 group was still higher than the amount released in the control hearts (Figure 10). This may explain the failure of KB-R7943 to rescue the mechanical performance of the heart in the 5-min Ca^2+^ depletion group. In addition, we cannot rule out the possibility that other calcium channels, i.e., the canonical transient receptor potential channels [35] may contribute to Ca^2+^ overload in the Ca^2+^ paradox.

In this study, we found that MDL28170 did not affect the release of troponin I from the Ca^2+^ paradoxic hearts, supporting the notion that activation of calpain mediates tissue death, but not contractile dysfunction. In addition, the ubiquitin proteasome system has been reported to mediate sarcomere degradation [40]; thus, further studies are needed to clarify whether this system participates in the Ca^2+^ overload-induced troponin I release. Figures 1 and 4 show that the area of surviving tissue in the 5-min Ca^2+^ depletion with MDL28170 group was smaller than the area in the corresponding 3-min Ca^2+^ depletion group. We suggest that the disruption of the sarcolemmal membrane caused by contracture aggravates calpain-mediated tissue death.

These results are clinically relevant because left ventricular diastolic dysfunction with a normal ejection fraction currently represents 40% to 50% of all heart failure cases [41], 44% to 75% in patients operated for coronary artery disease or aortic stenosis [42]. The left ventricular diastolic dysfunction is linked to an increased incidence of mortality and higher healthcare expenditures. Clinical and laboratory studies showed that loss of Ca^2+^ homeostasis and the subsequent development of contractures are some of the important factors for diastolic dysfunction [3], [4], [43]. Coincidently, calpain activated by Ca^2+^ overload has been implicated as an target for treating myocardial ischemia/reperfusion injury [18], [19]. In this study, we suggest that blocking both the development of diastolic dysfunction and the activation of calpain concurrently may be a therapeutic approach in limiting myocardial injury, especially in heart failure and cardiac surgery, and improving the survival of patients.

In summary, the present study provides the first evidence suggesting that the contracture is the main cause for contractile dysfunction, while activation of calpain mediates tissue death in the Ca^2+^ paradox. Further studies should focus on unfolding the molecular mechanism underlying the development of the contracture in the Ca^2+^ paradox.
